# American Spinal Injury Association (ASIA) Impairment Scale Motor Grade Outcomes Among Patients With Ballistic Neurologic Injuries Managed Expectantly: A 13-Year Single Institution Retrospective Review and Narrative Review of the Literature

**DOI:** 10.7759/cureus.107416

**Published:** 2026-04-20

**Authors:** Tyler X Giles, Thomas R Hemphill, Nicholas P Derrico, Rebekah Kimball, Benjamin Carroll, Katherine E Baker, Evan C Bowen, John Wilkinson, Gregory R Vance, Zachary S Smalley, Chad W Washington, Jared J Marks

**Affiliations:** 1 Neurosurgery, University of Mississippi Medical Center, Jackson, USA; 2 Radiation Oncology, University of Mississippi Medical Center, Jackson, USA

**Keywords:** gunshot wounds, natural history, neurotrauma, non-surgical management, outcomes

## Abstract

Background

Gunshot wounds (GSW) are increasingly prevalent in the United States. There is a paucity of data and guidelines governing surgical versus non-surgical management of neurologic GSW. One challenge lies in characterizing and stratifying the natural history of GSW to the nervous system (i.e., neurologic injuries) with respect to outcomes. Our group hypothesized that nonsurgical management would show promise and that rates of recovery would differ based on the injured area.

Methods

We reviewed ballistic nervous system injuries involving the spine and extremities over a 13-year period at an urban level 1 trauma center. We classified neurologic injuries by localization and motor grade using the American Spinal Injury Association (ASIA) grading system. We then defined the “ASIA shift” (i.e., the change from one motor grade to another) for each injury population to determine outcomes in ballistic injuries managed expectantly.

Results

Cervical spinal cord injuries (SCI), cauda equina injuries, and upper and lower extremity peripheral nerve injuries saw statistically significant improvement from non-antigravity to antigravity motor function while the L1 and conus medullaris injuries did not. Thoracic SCI had a higher rate of poor-grade injuries than other categories and saw the lowest rate of improvement. Cauda equina injuries saw significant improvement even when the injury implicated the spinal canal.

Conclusion

Our retrospective cohort study contributes to characterizing the natural history of GSW to the nervous system with respect to motor outcomes. Our data suggests a favorable course for select GSW-associated SCI and cauda equina injury patients managed without intervention.

## Introduction

The burden of ballistic injuries in the U.S. has been on the rise. The COVID-19 pandemic (defined as March 1, 2020, to February 28, 2021) was associated with a 15.0% increase in firearm-related incidents, a 34.3% increase in firearm-related nonfatal injuries, and a 28.4% increase in firearm-related deaths compared to pre-pandemic rates (defined as January 1, 2016, until the onset of the pandemic) [1]. The US Surgeon General, Dr. Vivek Murthy, declared firearm violence to be a public health crisis in 2024 [2]. As firearm injury rates have risen, so has healthcare expenditure. In 2020, the total healthcare cost of firearm-related injuries in the United States in 2020 was $493.2 billion, and there were an estimated 99,801 nonfatal firearm injuries [3]. For a Trauma Level I center in New Orleans, 2016-2019 brought 2,094 firearm injuries, which cost the hospital approximately $37 million and resulted in a net loss of $20 million [4]. A separate study in Los Angeles found that 1978-1992 brought 34,893 firearm injuries, which led to costs of $264 million ($606 million in 2026 dollars), 96% of which was borne directly or indirectly by public funds [5]. A 2005-2015 retrospective study found that government programs covered 41.6% of the cost burden of firearm-related admissions for 317,479 injuries, totaling $8.65 billion in healthcare expenses [6].

Management of patients with spinal column or neurological involvement from gunshot wounds (GSW) remains controversial. The literature is consistent that the presence of cerebrospinal fluid (CSF) fistulae, progressive neurological deficit, or spinal column instability are indications for surgery [7-12]. Neurologic deficits have been reported to be present in 33% - 92.4% of patients with spinal GSW [13]. Incomplete spinal cord injuries (ISCI), intoxication by the metal from the ballistics, and risk of ballistic fragment migration have also been noted as surgical indications [11,12]. Surgical decompression for patients with stable deficits is more controversial, and both surgical and nonsurgical management strategies are practiced widely [14]. Neurologic outcomes with respect to motor and functional outcomes among patients managed surgically have been reported with mixed results [15].

Nonsurgical management has shown promise in other studies. A comprehensive review of both civilian and military literature by Klimo et al. in 2010 found that, although the data were inconclusive, evidence did not support that decompressive surgery offered benefit in terms of neurological recovery [7]. Another systematic review published in 2013 by Sidhu et al. also did not find any evidence that operative intervention improves neurological outcome, aside from select patients with static compression and a progressive deficit [9]. In 2022, Platt et al. performed a meta-analysis for ballistic injuries to the lumbosacral spine which found that rates of neurological improvement were 72.3% after surgery and 61.7% with nonsurgical therapy, a difference that was not statistically significant [16]. In the same year, Goh et al. published a retrospective cohort study of 961 patients, 189 of whom were treated surgically [17]. They quoted a statistically significant improvement in the surgical cohort overall; however, subgroup analysis showed no benefit for cervical injuries and no improvement of lumbar complete injuries. Notably, there was no discussion of postoperative complications, which have been documented to be higher in surgery for these patients [15,18,19]. Given the risks and costs of surgery, it is vital to determine appropriate treatment paradigms. In this regard, a 2017 study found that for SCI associated with GSW, the surgical overutilization rate was 14.1% using surgical criteria commonly accepted in the literature (instability, T12-sacrum intracanalicular bullets, persistent CSF leak, neurologic decline or subcutaneous symptomatic bullets) [8]. Yet there is a paucity of data to demonstrate superiority of surgical intervention broadly; hence, there remains no consensus or guideline directives based on these operative indications, and much is left to the discretion of the treating physicians.

For any surgical intervention, it must have a favorable risk-to-benefit profile, and it should be expected to lead to improvement over the natural history of the disease process. We reviewed ballistic nervous system injuries involving the spine and extremities occurring over a 13-year period at a level 1 trauma center. The vast majority of patients in this period were managed without acute surgical decompression. We hope these data will help to more clearly define the natural history of such injuries to both set the expectations for recovery and establish benchmarks for future interventions. Our group hypothesized that nonsurgical management would show promising rates of recovery. Additionally, we hypothesized that rates of recovery would differ depending on the afflicted area.

## Materials and methods

We queried the neurosurgical trauma database at our institution for ballistic injuries involving the spine or extremities with an attributable neurologic deficit. We included all patients age ≥18 years old with ballistic neurologic injuries of the spine or peripheral nerves, which totaled 614 patients from 2010 to 2023. We then excluded patients who lacked follow-up motor exams, leaving 287 patients who had a ballistic nervous system injury and at least one follow-up visit. We classified neurologic injuries by localization and motor grade. We used the American Spinal Injury Association (ASIA) grading system - a standard and validated scale to classify SCI - to define grades [20-22]. Due to the variable degree and presentation of peripheral nerve injuries (brachial plexus, cauda equina, peripheral nerve injuries, etc.) and for consistency in our motor grade reporting, we extrapolated the ASIA grading scale to describe motor grades for these injuries as well (Table 1). 

**Table 1:** American Spinal Injury Association Impairment Scale (AIS) Generalized to Both Spine and Peripheral Nerves [22]

Grade	Injury Status	Description
A	Complete	No motor or sensory function is preserved in the sacral segments S4–S5 or peripheral nerve.
B	Incomplete	Sensory function but not motor function is preserved below the neurological level of the peripheral nerve or spine, including the sacral segments S4–S5.
C	Incomplete	Motor function is preserved below the neurological level of the spine or peripheral nerve, and more than half of key muscles below the neurological level have a muscle grade less than 3.
D	Incomplete	Motor function is preserved below the neurological level of the spine or peripheral nerve, and at least half of key muscles below the neurological level have a muscle grade of 3 or more.
E	Normal	Motor and sensory function are normal in the spine or peripheral nerve.

Given that motor outcomes appear to have the greatest bearing on functional recovery and quality of life in patients with spinal cord injuries, we chose to focus on motor grading as the primary outcome [23]. We extracted ordinal ASIA Impairment Scale (AIS) grades from documented patient exams. Inpatient and follow-up motor exam reporting was variable with respect to specific motor group scores, but all patients had their overall AIS grades reported. After collecting exam data from initial and follow-up visits, we then defined the “ASIA shift” (i.e., change from one motor grade to another) for each injury population, which was determined by comparing admission and follow-up exams. The generalized ASIA grading scale (Table 1) was used to determine AIS grades and motor function for peripheral nerves. We then stratified patients into two groups based on their ASIA shift - those with non-antigravity exams (ASIA A-C) and those with antigravity exams (ASIA D-E) - to assess meaningful functional improvement at follow-up. We also reviewed admission computed tomography imaging to assess spinal canal involvement and correlation with neurologic outcomes. We defined canal involvement as any degree of bony disruption of the cortical boundaries of the canal or bony or ballistic fragments visualized within the canal. AIS Grades and ASIA shift were determined by two neurosurgery residents who had completed junior residency and their major emergency room call experience. Comparison of initial and follow-up neurologic exams was performed using McNemar’s test with continuity correction using Excel (Microsoft, Redmond, WA, USA) and GraphPad/Prism (GraphPad Software, La Jolla, CA, USA), with groups stratified based on non-antigravity (ASIA A-C) and antigravity (ASIA D-E) motor status. IRB approval was received from our institution’s review board, including waiver of consent (UMMC-IRB-2023-329). This study was performed in accordance with the World Medical Association Declaration of Helsinki and all relevant laws and institutional guidelines. Privacy of human subjects was observed. This study was also performed in accordance with EQUATOR Strengthening the Reporting of Observational Studies in Epidemiology (STROBE) guidelines.

## Results

**Figure 1:**
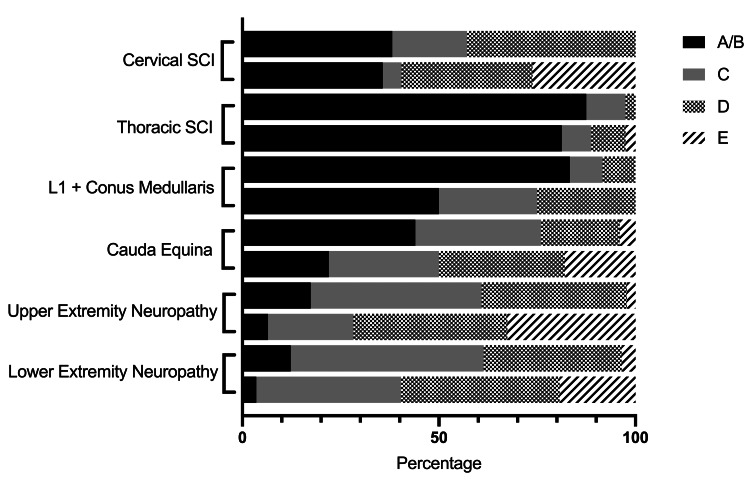
American Spinal Injury Association (ASIA) shift by neurologic injury from initial to follow-up. SCI: spinal cord injury

**Figure 2:**
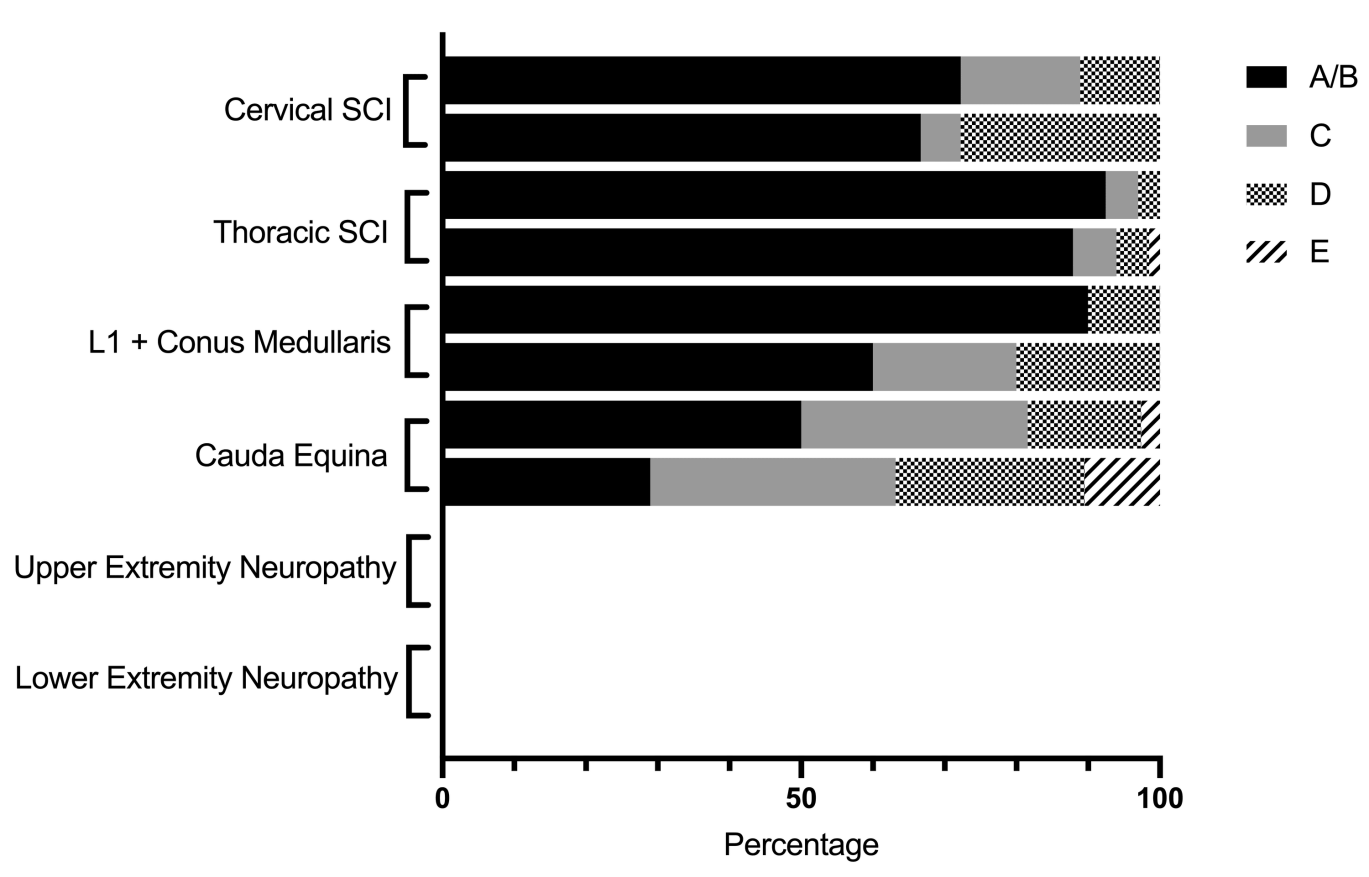
American Spinal Injury Association (ASIA) shift by neurologic injury with canal involvement from initial to follow-up. SCI: spinal cord injury

Of the 287 patients that had documented follow-up, our cohort had 42 (14.6%) cervical SCIs, 80 (27.9%) thoracic SCIs, 12 (4.2%) L1 or conus medullaris injuries, 50 (17.4%) cauda equina injuries, 46 (16.0%) upper extremity peripheral nerve injuries (including radiculopathy, plexopathies, and peripheral neuropathies), and 57 (19.9%) lower extremity peripheral nerve injuries (Table 2). Canal involvement was predominant in certain categories. Ten of 12 (83.3%) conus medullaris injuries, 66/80 (82.5%) thoracic SCIs, and 38/50 (76.0%) cauda equina injuries implicated the canal, compared to only 18/42 (42.9%) in the cervical SCIs. Patients with thoracic SCIs saw the lowest rate of improvement, with 8.8% (7/80) achieving ASIA D or E at follow-up, and patients with upper extremity peripheral nerve injuries saw the highest at 32.6% (15/46). Though patients in the cervical SCI group did have a 16.7% (7/42) ASIA shift from non-antigravity to antigravity exams, this was the only group that had no patients improve two or more grades in ASIA grade. All patients either had stable or improved motor exams at follow-up except for two patients with isolated peripheral nerve injuries occurring outside of the spine (Figures 1, 2). The change from non-antigravity to antigravity exams was statistically significant for all locations of injury except for conus medullaris SCI, which was limited by low sample size. Interestingly, patients with cauda equina injuries had significant improvement when the spinal canal was implicated in the injury (Table 2).

**Table 2:** American Spinal Injury Association (ASIA) Shift by neurologic injury from initial to follow-up with percentages and significance. *p<0.05 SCI: spinal cord injury

			Total (n)	Total w/ Follow-up (n)	w/ Follow up and Canal Involvement (n)	Initial		Follow-up		Improvement		McNemars A-C vs. D-E p-value
						ASIA A-C	ASIA D-E	ASIA A-C	ASIA D-E	*Δ ASIA D-E* *(absolute %)*	*ASIA Improvement 2+ grades* *(absolute %)*	
Cervical SCI	Intraspinal	CNS	51	42 (14.6%)	18 (42.9%)	24 (57.1%)	18 (42.9%)	17 (40.5%)	25 (59.5%)	16.7%	0 (0.0%)	0.023381*
Thoracic SCI			99	80 (27.9%)	66 (82.5%)	78 (97.5%)	2 (2.5%)	71 (88.8%)	9 (11.3%)	8.8%	6 (7.5%)	0.023381*
L1 + Conus Medullaris Injury			13	12 (4.2%)	10 (83.3%)	11 (91.7%)	1 (8.3%)	9 (75.0%)	3 (25.0%)	16.7%	2 (16.7%)	0.4795
Cauda Equina Injury		PNS	68	50 (17.4%)	38 (76.0%)	38 (76.0%)	12 (24.0%)	25 (50.0%)	25 (50.0%)	26.0%	14 (28.0%)	0.000873*
Upper Extremity Neuropathy	Extraspinal		79	46 (16.0%)	0 (0.0%)	28 (60.9%)	18 (39.1%)	13 (28.3%)	33 (71.7%)	32.6%	7 (15.2%)	0.0003*
Lower Extremity Neuropathy			92	57 (19.9%)	0 (0.0%)	35 (61.4%)	22 (38.6%)	23 (40.4%)	34 (59.6%)	21.0%	6 (10.5%)	0.00149*

Patients in this cohort were rarely managed surgically. There were 10 stabilizations for spinal column instability, two laminectomies, one acute repair of brachial artery injury with opportunistic exploration of a brachial plexus injury, and four delayed peripheral nerve explorations for neurolysis due to failure to improve. Hence our rate of surgical utilization was 5.92% (17/287). Our follow-up rate among the entire 614 patients considered was 46.7% (287/614) with an average follow-up time of 10.29 months. 

## Discussion

Most patients, regardless of injury location, presented with motor impairment consistent with ASIA grade A-C. The cervical spine was the most susceptible to blunt SCI when there was no canal involvement disclosed on computed tomography. Thoracic SCI, L1 and conus medullaris injuries, and cauda equina injuries occurred primarily alongside canal involvement, yet the thoracic and cauda equina injuries still demonstrated improvement. Cauda equina injuries saw the highest rate of improvement in 2+ ASIA grades. L1 and conus medullaris injuries saw similar rates of 2+ grade improvement when the spinal canal was involved.

A study of GSWs and blunt trauma at a level 1 trauma center conducted in 2024 found that 58.9% of incomplete GSW patients improved in their ASIA grade, even though only 12.2% received surgery. 81.4% of these surgeries were bullet removals. Ninety-four of 153 (61.4%) of cervical, 81/306 (26.5%) of thoracic, and 35/81 (43.2%) of lumbar GSW patients improved in neurological status. Twelve of 153 (7.8%) of cervical, 2/306 (0.1%) of thoracic, and 8/81 (9.9%) of lumbar GSW patients declined in status. Within the complete spinal GSW injuries, the median ASIA motor grade change was 0 (0.2 interquartile range (IQR)); within the incomplete injuries, the median ASIA motor grade change was 3 (0.12 IQR) [24]. A 2020 study of 21 patients with spinal GSWs found that 12 of 21 patients improved with an average increase of 1.17 in ASIA grade. Fourteen patients were managed without surgery, of which eight (57.1%) improved, and seven were managed surgically, of which four (57.1%) improved. Among all patients, only two patients improved two grades while the other 10 improved one grade. Thoracic injuries resulted in two follow-up deaths and a recovery rate of 3/11 (27.3%), while the lumbar spine saw all six patients improve with a mean increase of 1.3 in ASIA grade. One of those two deaths was due to sepsis and multiple organ failure and the other was due to pulmonary embolism. Among the non-surgically managed group alone, 3/3 lumbar and 3/3 cervical patients improved while only 2/8 thoracic patients improved [25].

Ge et al. in 2021 found that among 51 patients with GSWs that 70.6% of patients did not improve in ambulatory status while 21.6% did. Of these 51 patients, 26 (68.4%) were ASIA A, three (7.9%) were ASIA B, seven (18.4%) were ASIA C, and two (5.3%) were ASIA D. Within this study, 8/51 (15.7%) of patients were managed non-operatively [15]. For the cauda equina specifically, a 2023 study found that of 27 patients with primarily high-energy ballistic spine injuries, neurological deficits improved in 82.6% of patients managed operatively (88.9% of all study patients with 51.9% of surgeries performed within 24 hours of admission). All surgical patients had projectiles or fragments of bone or disc within the canal and underwent decompression and removal of the projectile or fragments, and, if deemed unstable, arthrodesis was performed. They did not report any surgical interventions for instability alone. Cauda equina syndrome was found to remain after one year in two patients; four patients developed painful neuropathies. In terms of ASIA grade, 7.4% of patients were graded an A; 3.7% a B; 48.14% a C; 22.2% a D and 18.5% were graded an E [26]. Lastly, a review from Klimo et al. in 2010 documents expected recovery from surgery from GSW for civilians [7]. Within this review, Benzel et al. in 1987 and Kihtir et al. in 1991 found 0% rates of improvement in complete SCI (CSCI) without operative management while Kupcha et al. in 1990 found the rate to be 50% [27-29]. Within ISCI, Kihtir found the rate of improvement without operation to be 0% while Kupcha and Benzel found it to be much higher, 60% and 75%, respectively. From our narrative review of current literature (Table 3), few papers discussed changes in ASIA grades after GSW by category and none discussed it by meaningful improvement in motor function with respect to antigravity status. Hence, we were unable to compare the findings of these papers directly to our methodology, and in this way our approach is novel.

**Table 3:** Comparison of current literature on improvement rates of spinal cord injuries (SCI) due to gunshot wounds and trauma. NR: Not Reported IQR: Interquartile Range CSCI: Complete Spinal Cord Injury ISCI: Incomplete Spinal Cord Injury ASIA: American Spinal Injury Association

Study	Number of Patients	Mean Follow Up	Patients with Improvement (%)	Improvement Details
Jacob et al., 2024 [24]	566	NR	58.9%	Median ASIA motor score change of 3 (0.12 IQR) with 12.2% operation rate
Nagy, Elgaidi, 2020 [25]	21	17 +/- 2.2 months	57.1%	1.17 avg. grade improvement in ASIA Score with 66% operated on. Among conservatively managed: 3/3 and 3/3 cervical and lumbar improved, 2/8 thoracic improved
Ge et al., 2021 [15]	51	50.4 +/- 46.8 months	21.6%	15.7% operation rate for all patients
Kessely et al., 2024 [26]	27	Follow-up at 3, 6, and 12 months	44.4% at 3 months, 59.3% at 6 months, 70.4% at 12 months	NR
Benzel et al., 1987 [27]	35 (8 cervical, 21 thoracic, 6 lumbosacral)	NR	0% of CSCI and 75% of ISCI improved w/o operation	20/35 (57.1%) were CSCI; 13/35 (37.1%) operation rate- all laminectomies
Kihtir et al., 1991 [28]	21 (7 thoracic, 14 lumbosacral)	3 months	0%	11/21 (52.4%) were CSCI; 0% operation rate
Kupcha et al., 1990 [29]	28 cervical	46 months	50% CSCI and 60% of ISCI w/o operation improved	21/28 (75%) were CSCI; 5/28 (17.9%) operation rate- all laminectomies
Our study, 2026	287	10.9 months	40.2%	17/287 (5.9%) operation rate

These data suggest an expected benchmark for recovery from spinal cord GSW with respect to motor grades along with the natural history of various ballistic injuries, which was not previously reported. Based on our results, the rate of necessary surgical stabilization was 5.92% (17/287), which is slightly higher than the current literature. The prevailing indication for stabilization was concern for instability and deformity demonstrated on CT or when challenged with upright plain films. One stabilization was performed on a fracture that was otherwise anticipated to be braced, but ultimately required operative CSF leak repair for a subarachnoid-pleural fistula and was stabilized due to heightened risk of iatrogenic instability. There were no delayed surgical stabilizations found, which may suggest surgery could have been deferred in some patients. We performed two laminectomies among the reported patients. One patient had a retained intracanalicular bullet and trap-door laminoplasty was performed to remove the bullet for prevention of lead toxicity and ongoing neural compression - this patient had a ballistic cauda equina injury and improved from ASIA D to E. The other patient had a retained thoracic bullet fragment in a lower thoracic neural foramen and underwent lower thoracic laminectomies after experiencing a delayed but abrupt decline (from ASIA D to A) in lower extremity strength during hospital stay. A compressive epidural hematoma was found and removed with prompt improvement in post-operative strength back to ASIA D. There were no other surgical decompressions in our cohort. Consistent with our experience, a 2009 study of 4,204 patients found that spinal instability after a GSW to the head, neck, or torso is very rare. None of the 4,204 patients with GSW to the head, neck, or torso demonstrated spinal instability requiring surgical intervention and 0.6% (2/327) of patients required any type of operative decompression [30]. A 2011 paper similarly found that the rate of instability in the cervical spine following a penetrating neck injury was extremely low at 0.4%. For a GSW specifically, it was less than 1% [31]. 

Limitations of our study include single institution review and sample size restrictions due to low follow-up rate, which may have confounded results. Additionally, the types of firearms that caused these injuries were not reported. However, we expect that most were handguns, given that our cohort was treated at a civilian level 1 trauma center, where it has been shown that most GSW are caused by handguns [32]. Our follow-up rate of 46.7% (287/614) is in keeping with other published studies that report lost to follow-up rates between 41.4% and 69% for gun violence survivors [33,34]. However, this follow-up rate may still skew our results towards patients with better recovery. In addition, the variability in the timing for follow-up exams limits and minimal information on rehabilitation limits consistency. The extrapolation of the ASIA grades to peripheral nerves is also novel and therefore not previously validated. Further validation with other cohorts presents opportunities for additional research. Further studies could also delve deeper into quality of life issues such as sphincter control or pain. However, given a lack of need for delayed surgical stabilization among our patient population, these reports seem to confirm that the rate of true delayed instability is likely lower than our rate of surgical stabilization. 

## Conclusions

Understanding the natural history of spinal cord and peripheral nervous system ballistic injuries can aid in discussions with patients and families and affirm when the decision is to manage expectantly. Such evidence may contribute to the development of future guidelines. We report the experience of a civilian regional trauma center with ballistic nervous system injuries to the spine and extremities over 13 years. All injury categories except L1 and conus medullaris injuries saw significant improvement from non-antigravity to antigravity motor function. Although L1 and conus medullaris injuries did not have statistically significant ASIA shift, analysis was hindered by small sample size and some of the patients did improve to antigravity. Cauda equina injuries saw significant improvement even when the canal was involved. Despite some reports of high rates of surgical intervention for decompression in the setting of canal involvement, these data suggest a favorable course for select GSW-associated SCI and cauda equina injury patients managed without intervention. Additionally, in agreement with prior reports, surgical intervention for true delayed instability or deformity appears to be rarely indicated.
